# Improving the Effectiveness of Exercise Therapy for Adults With Knee Osteoarthritis: A Pragmatic Randomized Controlled Trial (BEEP Trial)

**DOI:** 10.1016/j.arrct.2023.100266

**Published:** 2023-05-06

**Authors:** Nadine E. Foster, Elaine Nicholls, Melanie A. Holden, Emma L. Healey, Elaine M. Hay, The BEEP trial team

**Affiliations:** a STARS Education and Research Alliance, Surgical Treatment and Rehabilitation Service, The University of Queensland and Metro North Health, Brisbane, Australia; b Primary Care Centre Versus Arthritis, School of Medicine, Keele University, Keele, UK; c Keele Clinical Trials Unit, Keele University, Keele, UK.

**Keywords:** Exercise, Function, Knee, Osteoarthritis, Pain, Randomized controlled trial, Rehabilitation

## Abstract

**Objective:**

To investigate whether knee osteoarthritis (OA) related pain and function can be improved by offering enhanced physical therapist-led exercise interventions.

**Design:**

Three-arm prospectively designed pragmatic randomized controlled trial.

**Setting:**

General practices and National Health Service physical therapy services in England.

**Participants:**

514 adults (252 men, 262 women) aged ≥45 years with a clinical diagnosis of knee osteoarthritis (N=514). Mean Western Ontario and McMaster Universities Osteoarthritis Index (WOMAC) scores at baseline were 8.4 for pain and 28.1 for function.

**Interventions:**

Participants were individually randomized (1:1:1 allocation) to usual physical therapy care (UC control: up to 4 sessions of advice and exercise over 12 weeks), individually tailored exercise (ITE: individualized, supervised, and progressed lower limb exercises, 6-8 sessions over 12 weeks), or targeted exercise adherence (TEA: transitioning from lower limb exercise to general physical activity, 8-10 contacts over 6 months).

**Main Outcome Measures:**

Primary outcomes were pain and physical function measured by the WOMAC at 6 months. Secondary outcomes were measured at 3, 6, 9, 18, and 36 months.

**Results:**

Participants receiving UC, ITE, and TEA all experienced moderate improvement in pain and function. There were no significant differences between groups at 6 months (adjusted mean differences (95% confidence intervals): pain UC vs ITE, -0.3 (-1.0 to 0.4), UC vs TEA, -0.3 (-1.0 to 0.4); function UC vs ITE, 0.5 (-1.9 to 2.9), UC vs TEA, -0.9 (-3.3 to 1.5)), or any other time-point.

**Conclusions:**

Patients receiving UC experienced moderate improvement in pain and function; however, ITE and TEA did not lead to superior outcomes. Other strategies for patients with knee osteoarthritis to enhance the benefits of exercise-based physical therapy are needed.

## Highlights

• Exercise is recommended for knee OA but effects are small and reduce over time.
• This randomized trial compared 3 physical therapist-led exercise interventions.
• 514 adults with knee OA took part, with data collected over a 36-month follow-up.
• On average, all 3 groups experienced moderate improvement in pain and function.
• There were no significant differences between groups, at 3, 6, 9, 18, and 36 months.

Exercise, including local muscle strengthening exercise and general aerobic fitness, is recommended as “core” treatment for individuals with knee osteoarthritis (OA).^1^ Although systematic reviews conclude that exercise is more effective than non-exercise treatment in reducing pain and improving physical function,^2^, ^3^, ^4^ the average effects are modest and decline over time, potentially explained by diminishing exercise adherence. It is unclear whether the effects of exercise for knee OA can be improved by changing the characteristics of the exercise program.

Systematic reviews highlight the importance of individualized exercise, regular exercise, supervision and follow-up, as well as educational and behavioral strategies to enhance exercise adherence.^2^^,^^3^^,^^5^^,^^6^ A previous randomized controlled trial (RCT) investigating physical therapy-led exercise for knee OA delivered over an average of 4 treatment sessions, showed that pain reduction was 3 times greater and functional improvement 4 times greater compared with standardized exercise advice.^7^ Benefits declined by 6 months follow-up. A further RCT tested a more intensive physical therapy-led exercise program (average of 6 treatment sessions) and showed greater improvements in pain than those observed in the previous trial.^8^ Exercise appears to be worth doing but trials are needed that test if outcomes can be improved through greater individualization, supervision and progression of lower limb exercise and whether the effects of exercise can be maintained for longer through changing the focus from lower-limb exercise to overall physical activity in order to improve adherence.^6^^,^^9^, ^10^, ^11^ The BEEP (Benefits of Effective Exercise for knee Pain) trial aimed to test whether knee OA related pain and function can be improved by offering these enhanced physical therapist-led exercise interventions.

## Methods

### Design

A 3 parallel-group, pragmatic RCT, prospectively registered with the International Standard of Randomized Controlled Trials Number Registry (ISRCTN 93634563), with embedded health economic evaluation and linked qualitative interviews (both reported separately).^12^^,^^13^ The trial was approved by the North West Research Ethics Committee in the UK (REC reference: 10/H1017/45). There were no substantial amendments to the methods of the trial. The full trial protocol was published previously.^14^

### Setting and participants

Participants were recruited from 65 general practices and 5 National Health Service (NHS) physical therapy services in the West Midlands and Cheshire regions of the UK. Adults aged ≥45 years with knee pain and/or stiffness who met the National Institute for Health and Care Excellence criteria for a clinical diagnosis of knee OA,^1^ and who were able to read and write in English, willing to participate, able to give full informed consent, and who had access to a telephone (for minimum data collection), were eligible. Patients were excluded if they had alternative diagnoses or serious underlying pathology (eg, inflammatory arthritis); total hip/knee joint replacement on the affected side, on a waiting list for a total knee/hip replacement; exercise interventions contra-indicated; received a physical therapist-led exercise program or injection into the painful knee in the last 3 months.

Based on the learning from a pilot study (ISRCTN 23294263),^14^ we identified potentially eligible participants in 3 ways: (1) general practice electronic record reviews to identify older adults who had consulted for knee pain in the last 12 months, (2) population survey of older adults registered with participating practices, and (3) older adults referred from general practice to physical therapy for knee pain. Individuals identified from methods (1) and (2) were mailed a brief screening questionnaire. Individuals identified from method 3 were first screened by a member of the physical therapy service team for key eligibility criteria. Those who were eligible and agreed to further contact were mailed trial information, then telephoned by a research nurse to check eligibility, discuss trial participation, and obtain written informed consent. No physical examination was conducted until trial participants’ first physical therapy appointment. We therefore anticipated that a small number of participants would be found to be subsequently ineligible.

### Randomization and masking

Following informed consent and receipt of the baseline questionnaire, a trial administrator randomized participants using a password protected computer-generated randomization schedule provided by the Clinical Trials Unit. This ensured that research nurses and trial statistician remained blind to treatment allocation. Participants were individually randomized to 1 of 3 treatment groups with a 1:1:1 allocation ratio using random permuted blocks of size 3, stratified by physical therapy clinic. Physical therapists and participants could not be blinded to allocation but research nurses responsible for data collection were blinded to allocation. The statistician remained blind until analysis of 18-month follow-up data (analysis of 36-month data were conducted after unblinding).

### Interventions

Treatment was delivered in 5 NHS physical therapy services by 47 BEEP trained physical therapists across 10 treatment clinics. Each physical therapist was trained to deliver 1 of the 3 interventions. Full details of the interventions and the differences between them have been published previously,^14^ as has the content and evaluation of the physical therapy training programmes.^15^ Standardized case report forms were used to record treatment details.

All patients received a BEEP trial information booklet which included information about the value of exercise and physical activity and simple self-help messages. All patients were instructed in a home exercise program based on best practice guidance about exercise dose,^16^, ^17^, ^18^ and that guided participants to continue at home with the same exercise program as that prescribed by their physical therapist. All patients could continue to access usual care in addition to BEEP treatment.^14^ The content of the interventions is summarized briefly below.

#### Usual physical therapy care

Usual physical therapy care (UC) consisted of advice and lower limb exercise delivered in up to 4, individual, 1-to-1 treatment sessions over 12 weeks. Exercises were selected from an agreed template of commonly prescribed lower limb exercises (available from the authors on request), including muscle strengthening (non-weight-bearing and weight-bearing), range of movement, or stretching exercises. UC matched usual physical therapy practice in the NHS.^19^

#### Individually tailored exercise

Individually tailored exercise (ITE) consisted of a supervised, individually tailored and progressed lower limb exercise program delivered in 6-8 1-to-1 treatment sessions over 12 weeks. The exercise program focused on strengthening, stretching and balance exercise, and functional task training. Agreed and defined functional and exercise goals were reviewed and progressed. Individualization was based on physical therapist assessment findings, including biomechanical and physiological observations, pain responses to specific exercises and starting levels of strength, range of movement, and balance. Participants were given a print-out of their specific exercise prescription (using PhysioTools computer software), which changed over time as the exercise program progressed. Physical therapists encouraged exercise behavior change using self-monitoring via a lower limb exercise diary to record adherence.

#### Targeted exercise adherence

Targeted exercise adherence (TEA) began with a focus on lower limb exercise (as in the ITE protocol) but aimed to support a transition to increasing general physical activity adherence over 6 months. It included 4 individual face-to-face treatments up to week 12, and a further 4-6 follow-up contacts (face-to-face or over the telephone) from week 12 to 6 months (a total of 8-10 treatment contacts). The target by the end of 6 months was that participants would be engaged in physical activity opportunities within their community, having had support from their physical therapist to overcome initial problems or barriers in engaging in these activities. The emphasis was therefore on maintenance of physical activity beyond the period of support from the physical therapist.

In addition to prescribing an individualized, progressed, and supervised lower limb exercise program (as per ITE), physical therapists assessed participants’ current general physical activity levels, intentions to increase physical activity, attitudes to exercise for knee pain and general health, and individual barriers and facilitators to exercise. They also helped patients to identify suitable general physical activity opportunities in their local community. Each physical therapist was provided with an “adherence enhancing toolkit” that contained optional educational, behavioral, and cognitive-behavioral tools and techniques for facilitating exercise behavior change (for a summary of the contents of the toolkit, see additional file 4 in the BEEP trial protocol paper^14^). Specific tools were selected for use with individual patients based on assessment findings.

### Outcome measures and follow-up

Outcomes were measured via postal questionnaires, with reminders, at baseline, 3, 6, 9, 18, and 36 months follow-up. At 6, 18, and 36 months follow-up, minimum data were collected over the telephone by a blinded research nurse.

The primary outcomes were lower limb pain and function measured using the Western Ontario and McMaster Universities Osteoarthritis Index (WOMAC),^20^ 6 months post randomization. Secondary outcomes were the proportion of treatment responders (Outcome Measures in Rheumatology Clinical Trials-Osteoarthritis Research Society International [OMERACT-OARSI] clinical responder criteria);^21^^,^^22^ self-reported physical activity (Physical Activity Scale for the Elderly (PASE);^23^ self-reported use of local physical activity facilities in the previous 7 days (single item); exercise adherence (self-reported adherence to prescribed exercises); self-reported body mass index (calculated from self-reported height and weight); a modified version of a measure of treatment acceptability and credibility;^24^^,^^25^ illness perceptions (Brief Illness Perceptions Questionnaire);^26^ confidence in ability to exercise (Self-efficacy for Exercise Scale);^27^ outcome expectations for exercise (Outcome Expectations for Exercise Scale 2);^28^ anxiety (Generalized Anxiety Disorder Assessment 7);^29^ depression (Personal Health Questionnaire Depression Scale 8);^30^ self-reported health care resource use (both NHS and private health care); and overall health related quality of life (EQ-5D-3L),^31^ (for health economic analysis, reported separately^12^). Seven-day accelerometry was also measured in a subsample of participants (n=89) via Actigraph accelerometers (models: GT1M, GT3X, and GTX+).

### Sample size and power

A sample of 500 participants was required to detect an effect size of 0.35 for both WOMAC pain and function at 6 months follow-up,^32^ with 2-tailed testing, power of 80% and an alpha level of 5%, comparing UC with either ITE or TEA. This allowed for a 20% loss to follow-up. Standard deviations for WOMAC pain and WOMAC function at 6 months follow-up were drawn from a previous trial^8^ and estimated to be 5 and 17, respectively.

### Statistical analysis

The statistical analysis plan for the BEEP trial has been previously published.^14^ Briefly, primary and secondary treatment models were derived separately at each follow-up time-point by comparing ITE and TEA interventions to UC on an intention-to-treat basis, using analysis of covariance or logistic regression as appropriate within STATA (v15). Results were presented as mean or percentage differences, as appropriate, with 95% confidence intervals, after adjustment for age, sex, duration of the knee problem, physical therapy treatment clinic, and the baseline score of the outcome of interest and after missing data were imputed using previously used methods^33^ (described in detail in appendix 1). Sensitivity analyses were performed, including per protocol analysis; adjusting treatment models for therapist effects; excluding *a priori* covariates from the analysis; and not imputing missing data. Accelerometry data were analyzed using the same methods as for the primary and secondary outcomes; however, missing data were not imputed.

The longitudinal trajectories of the WOMAC and PASE scores were modeled by treatment group using generalized estimating equations after adjusting for the *a priori* covariates previously defined. Exercise adherence was reported descriptively, and the 6-month treatment effects for WOMAC pain and function explored to see if treatment effect differed depending on exercise adherence. This analysis was conducted by including exercise adherence as a main effect, and as an interaction with treatment, in the models for the primary analysis.

## Results

### Participant flow and characteristics

**Fig 1:**
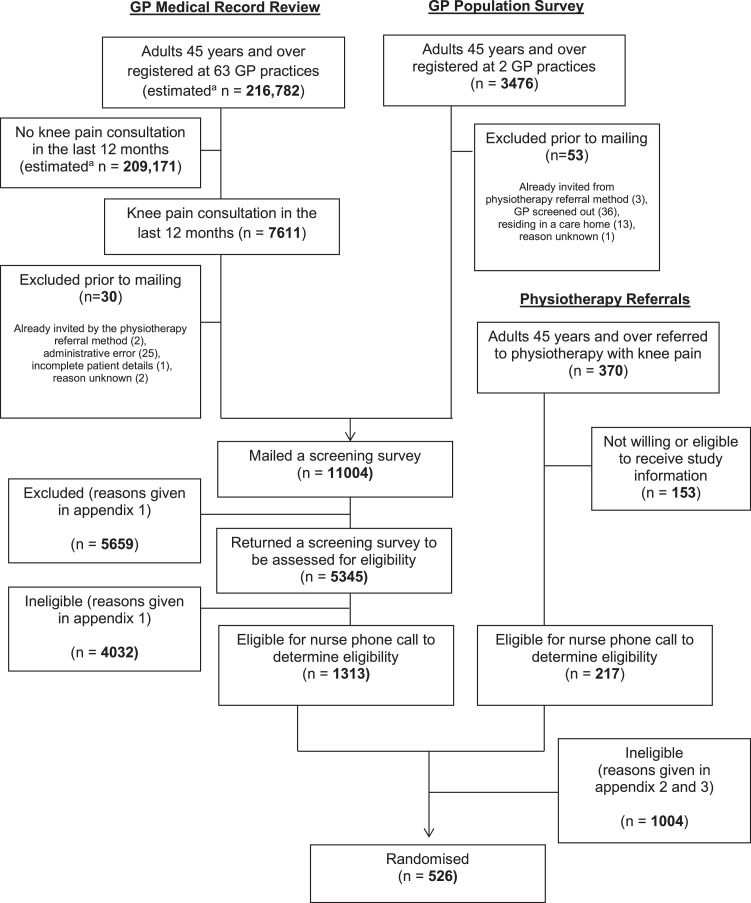

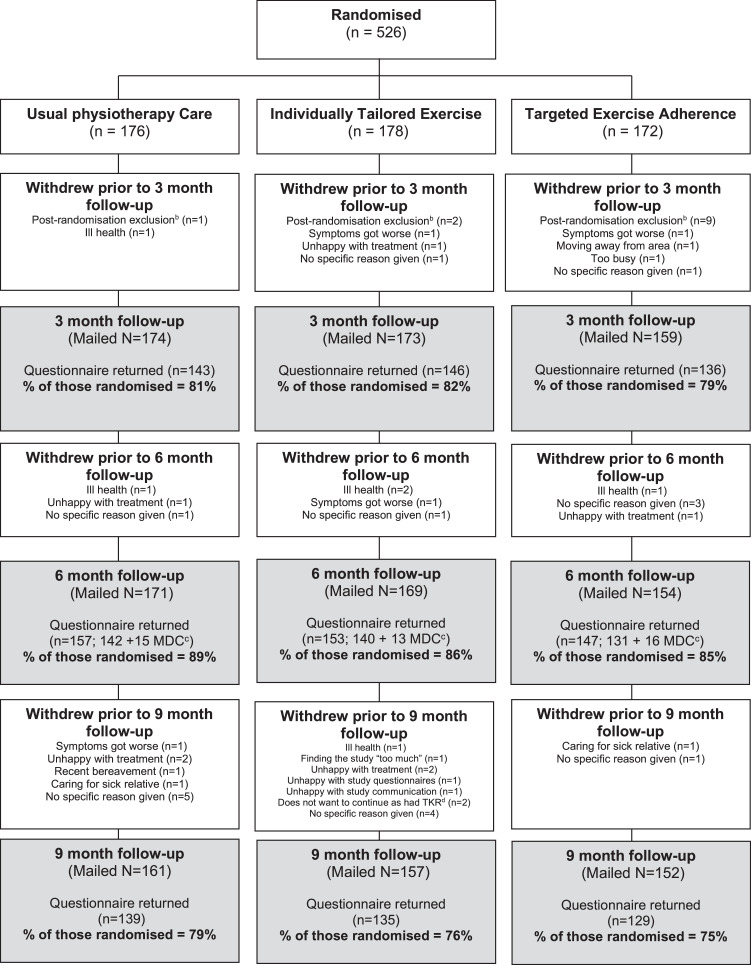

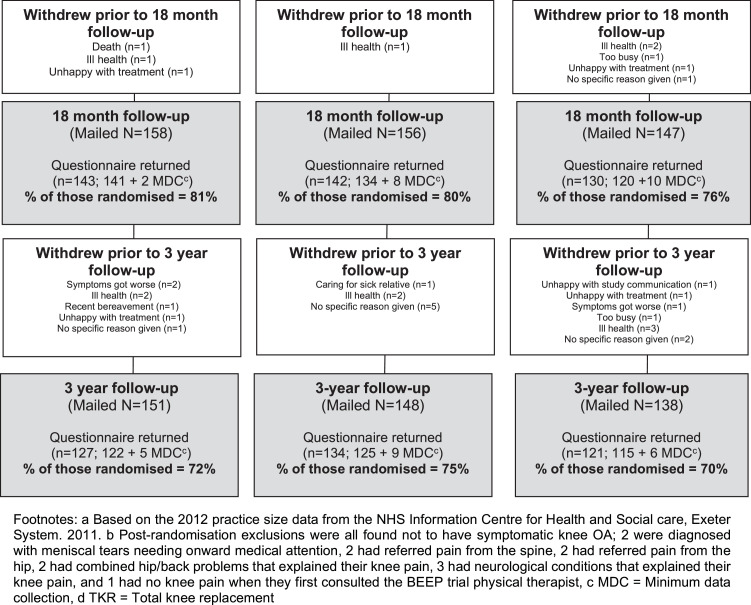
BEEP recruitment flow diagram.

**Fig 2:**
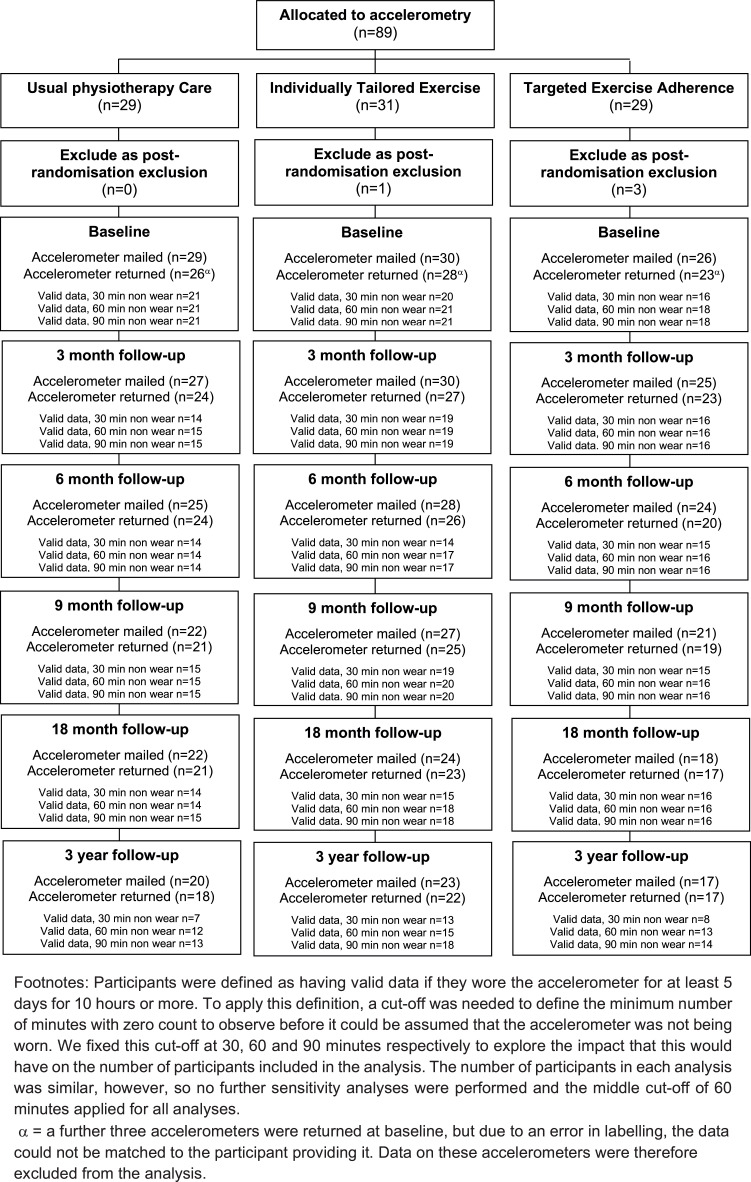
Flow chart of accelerometer allocation and return.

Figures 1 and 2 illustrate the flow of patients during the trial and recruitment to the accelerometer sub-sample. Of 1530 potentially eligible participants, 526 (34%) were randomized between October 2010 and February 2012 and followed up until 1st of April 2015. Twelve participants randomized were found to be subsequently ineligible at their first BEEP trial physical therapy assessment (see fig 1), and excluded from follow-up and analyses. Therefore, 514 participants form the dataset for the BEEP trial.

There were no important differences between groups at baseline (table 1). On average, participants had moderate pain and disability, symptom durations of between 1 and 5 years, were overweight and reported low physical activity levels, but were positive about the ability of treatment to help their knee problem and had generally positive expectations about the benefits of exercise.

**Table 1:** Baseline characteristics of participants (N=514). Values are means (standard deviations) unless otherwise stated

Key Characteristics	UC N=175	ITE N=176	TEA N=163	Overall N=514
Age (years)	62 (9)	63 (11)	64 (9)	63 (10)
Women sex*	87 (50)	91 (52)	84 (52)	262 (51)
Married/cohabiting*	135 (77)	134 (77)	126 (79)	395 (78)
Currently in a paid job*	81 (47)	67 (39)	66 (41)	214 (42)
Body-mass index (kg/m^2^)	29.5 (5.8)	29.4 (5.7)	30.0 (5.6)	29.6 (5.7)
Comorbidities				
Heart disease (including high blood pressure, angina, heart failure, stroke and heart attack)*	96 (55)	85 (49)	80 (49)	261 (51)
Lung disease (including asthma and bronchitis)*	28 (16)	36 (21)	24 (15)	88 (17)
Diabetes*	27 (15)	22 (13)	17 (10)	66 (13)
Depression*	40 (23)	47 (27)	27 (17)	114 (22)
Osteoporosis*	9 (5)	14 (8)	14 (9)	37 (7)
Pain in the last month				
Bilateral knees*^,^†	70 (40)	83 (47)	78 (48)	231 (45)
Upper limb*^,^†	80 (46)	77 (44)	67 (41)	224 (44)
Lower limb, excluding the knee*^,^†	121 (69)	139 (79)	118 (73)	378 (74)
Widespread pain*^,^†	25 (14)	30 (17)	24 (15)	79 (15)
WOMAC				
Pain (0-20)	8.2 (3.2)	8.5 (3.7)	8.5 (3.5)	8.4 (3.5)
Stiffness (0-8)	3.7 (1.7)	3.7 (1.9)	3.8 (1.6)	3.7 (1.7)
Function (0-68)	27.4 (12.0)	27.8 (12.7)	29.1 (12.0)	28.1 (12.2)
Onset of knee problem*				
In the last 12 months	39 (22)	46 (26)	42 (26)	129 (25)
>1 year but <5 years	74 (42)	60 (35)	67 (41)	200 (39)
>5 years but <10 years	33 (19)	37 (21)	26 (16)	98 (19)
>10 years	32 (18)	33 (19)	28 (17)	93 (18)
Currently taking medication for knee problem*^,^‡	127 (73)	123 (73)	118 (77)	368 (74)
PASE (0-590)	177 (81)	176 (87)	180 (83)	177 (84)
Previous personal experience of exercise on a regular basis*	115 (66)	102 (59)	101 (64)	318 (63)
Used local facilities or opportunities that involved any form of physical activity in the last 7 days*	63 (36)	56 (32)	47 (29)	164 (32)
Self-Efficacy for Exercise Scale (SEE) (0-10)	5.5 (2.3)	5.5 (2.3)	5.3 (2.5)	5.4 (2.3)
Outcome Expectations for Exercise Scale (OEE)				
Positive subscale (1-5)	3.9 (0.6)	3.9 (0.6)	4.0 (0.6)	3.9 (0.6)
Negative subscale (1-5)§	3.6 (0.8)	3.6 (0.9)	3.4 (0.8)	3.5 (0.8)
Generalized Anxiety Disorder Assessment (GAD-7) (0-21)||	2.0 (0.0, 5.0)	1.2 (0.0, 4.2)	2.0 (0.0, 4.0)	2.0 (0.0, 5.0)
Participant Health Questionnaire Depressive Scale (PHQ-8) (0-24)||	3.0 (1.0, 6.0)	3.0 (1.0, 5.6)	2.0 (0.8, 5.0)	2.2 (1.0, 5.7)
Brief illness perceptions questionnaire				
How much does your knee pain affect your life? (0-10)	5.5 (2.1)	5.5 (2.2)	5.5 (2.4)	5.5 (2.2)
How long do you think your knee pain will continue? (0-10)||	10.0 (7.0, 10.0)	9.9 (7.0, 10.0)	9.3 (7.0, 10.0)	10.0 (7.0, 10.0)
How much control do you feel you have over your knee pain? (0-10)	4.4 (2.7)	4.0 (2.5)	3.9 (2.8)	4.1 (2.7)
How much do you think treatment can help your knee pain? (0-10)||	7.0 (5.0, 8.2)	7.0 (5.0, 8.0)	7.0 (5.0, 9.0)	7.0 (5.0, 9.0)
How much do you experience symptoms from your knee pain? (0-10)	6.2 (1.9)	6.2 (2.1)	6.2 (2.3)	6.2 (2.1)
How concerned are you about your knee pain? (0-10)||	8.0 (6.0, 10.0)	8.0 (6.0, 10.0)	8.0 (6.8, 10.0)	8.0 (6.0, 10.0)
How well do you feel you understand your knee pain? (0-10)	5.7 (2.7)	5.9 (3.0)	5.3 (3.3)	5.7 (3.0)
How much does your knee pain affect you emotionally? (0-10)	4.7 (3.1)	4.9 (3.1)	4.9 (3.0)	4.8 (3.1)
Accelerometer data¶ (sub-sample of trial participants)				
N	21	21	18	60
Average# number of counts per minute (measured by accelerometers)||	311 (215, 390)	253 (177, 302)	232 (163, 299)	246 (180, 329)
Participants meeting physical activity guidelines*^,^⁎⁎	4 (19)	1 (5)	0 (0)	5 (8)

NOTE. All figures are based on data after multiple imputation of missing data has been applied (with the exception of the accelerometer data, comorbidity data, data on marital status, employment status, previous exercise experience, and pain at other body sites). WOMAC higher score=worse outcome; PASE higher score=more active; SEE higher score=more confident that exercise can be done; OEE positive and negative subscales higher score=higher expectations that exercise will be beneficial; GAD-7 higher score=more anxious; PHQ-8 higher score=more depressed; IPQR – affects life, higher score=more affected; IPQR – duration, higher score=lasts a longer time; IPQR – personal control, higher score=more control; IPQR – treatment control, higher score=higher belief treatment can control; IPQR – symptom experience, higher score=more symptoms that are more severe; IPQR – concern, higher score=more concerned; IPQR – understanding, higher score=more understanding; IPQR – emotion, higher score=more emotionally affected.

⁎ Numbers are N (percentage).

† Defined using the pain regions of the Manchester definition of widespread pain.^41^

‡ Includes both prescribed and over-the-counter medications.

§ Reverse scored that is, a higher score on the negative subscale indicates higher expectations of the benefit of exercise.

|| Median (interquartile range).

¶ Participants are only included if they have worn the monitor for at least 5 days for 10 hours or more. Valid time is calculated assuming that any consecutive runs of zero count lasting for 60 minutes or more are counted as non-wear.

# An average score was calculated for each participant by averaging the total number of counts across the valid time for which the accelerometer was worn.

⁎⁎ Defined as participants completing 150 minutes each week of moderate intensity physical activity (accumulated in bouts of 10 minutes or more) or 75 minutes of vigorous intensity activity spread across the week (adapted from Regnaux et al^39^). Bouts calculated using a drop-time of 2 minutes.^40^ Missing days of data are imputed using the average of the average count for days where data are present.

Primary outcome data were obtained from 457 (87%) of participants at 6 months (157 (89%) in UC, 153 (86%) in ITE and 147 (85%) in TEA, respectively) (fig 1). Participants lost to follow-up at 6 months had slightly worse baseline WOMAC knee pain and function scores and slightly higher levels of anxiety and depression at baseline than those who returned follow-up data. At baseline, they also were less likely to report having used local physical activity facilities or opportunities in the last 7 days.

### Treatment received and adverse events

The number of patients treated at each clinic and by each physical therapist in the BEEP trial is shown in appendix 2. Thirty-nine participants (8%) received no physical therapy sessions despite several contact attempts (UC n=12 (7%), ITE n=15 (9%), TEA n=12 (7%)). Participants in UC had fewer treatment sessions (median 3, interquartile range (IQR) 2, 4, range 0-8) than those in ITE (median 6, IQR 4, 6.5, range 0-9) and TEA (median 7, IQR 4, 8, range 0-11). In total, 156 (89%), 109 (62%), and 78 (48%) of the physical therapists’ case report forms within UC, ITE, and TEA, respectively, were judged to be per protocol. The treatment that participants received in each group is summarized in table 2.

**Table 2:** Summary of treatment from case report forms for participants attending at least 1 physical therapy session (n=475)

	UC	ITE	TEA
	N=163	N=161	N=151
*Data Obtained From All Case Report Forms*			
Number of treatment sessions provided (median; interquartile range)	3; 2, 4	6; 4, 6.5	7; 4, 8
Length of face to face treatment session (mean (standard deviation) duration in minutes)	33 (12)	31 (10)	31 (10)
Assessment/reassessment	160 (98)	161 (100)	151 (100)
Education and advice	160 (98)	160 (99)	149 (99)
Supervised exercise in clinic	141 (87)	159 (99)	142 (94)
Home exercises provided/reviewed	157 (96)	161 (100)	127 (84)
Physio tools exercise sheet	152 (93)	148 (92)	128 (85)
Prescribed lower limb/knee exercise*	155 (95)	161 (100)	149 (99)
Advice and treatment other than exercise†	37 (23)	53 (33)	35 (23)
*Data Only Obtained Within ITE Case Report Forms*			
Exercise progressed	-	140 (87)	-
Exercise diary provided/reviewed (including rating of perceived exertion)	-	119 (74)	-
*Data Only Obtained Within TEA Case Report Forms*			
Telephone contact in addition to face-to-face contact	-	-	94 (62)
Prescribed general exercise/physical activity‡	-	-	112 (74)
Adherence enhancing strategies from tool kit used			
*Educational aids*			
*Written educational aid*	-	-	130 (86)
*Behavioral aids*			
*Pedometer*	-	-	80 (53)
*Physiotools software*	-	-	128 (85)
*Visual feedback chart*	-	-	2 (1)
*Reminder postcard*	-	-	7 (5)
*Graded activity sheet*	-	-	22 (15)
*Physical activity/exercise diary (including rating of perceived exertion)*	-	-	118 (78)
*Monitoring heart rate*	-	-	9 (6)
*Cognitive behavioral aids*			
*Eliciting health-related beliefs*	-	-	54 (36)
*Identifying exercise/activity barriers/facilitators*	-	-	53 (35)
*SMART goal setting/contracting*	-	-	45 (30)
*Rulers (readiness, confidence, importance ruler)*	-	-	19 (13)
*Decisional balance sheet*			1 (1)
*Set back plan*	-	-	54 (36)
*Local lifestyle change opportunities*			
*Identifying local exercise and activity opportunities*	-	-	78 (52)

NOTE. Values are numbers and percentages unless otherwise stated.

⁎ Commonly muscle strengthening exercise, range of movement/stretching exercise, proprioception/balance exercise.

† Commonly advice about orthotics, ice therapy, and manual therapy.

‡ Commonly walking, cycling, or swimming. There was also evidence of individualization of the prescribed activities to participants’ preferences including, bowling, going to the gym, running, golf, Zumba, dancing, Pilates, football, and tennis.

No serious adverse events and 4 adverse events attributable to the interventions were reported; 1 in UC (sprained ankle), 2 in ITE (sprained ankle and twisted painful knee), and 1 in TEA (fall while walking). 82 participants experienced muscle soreness or transient increases in pain/aching (UC n=31 (19%), ITE n=33 (20%), TEA n=18 (12%)). Over the 3-year follow-up, there was 1 death, which was not attributable to the BEEP trial interventions.

### Primary and secondary outcomes

**Fig 3:**
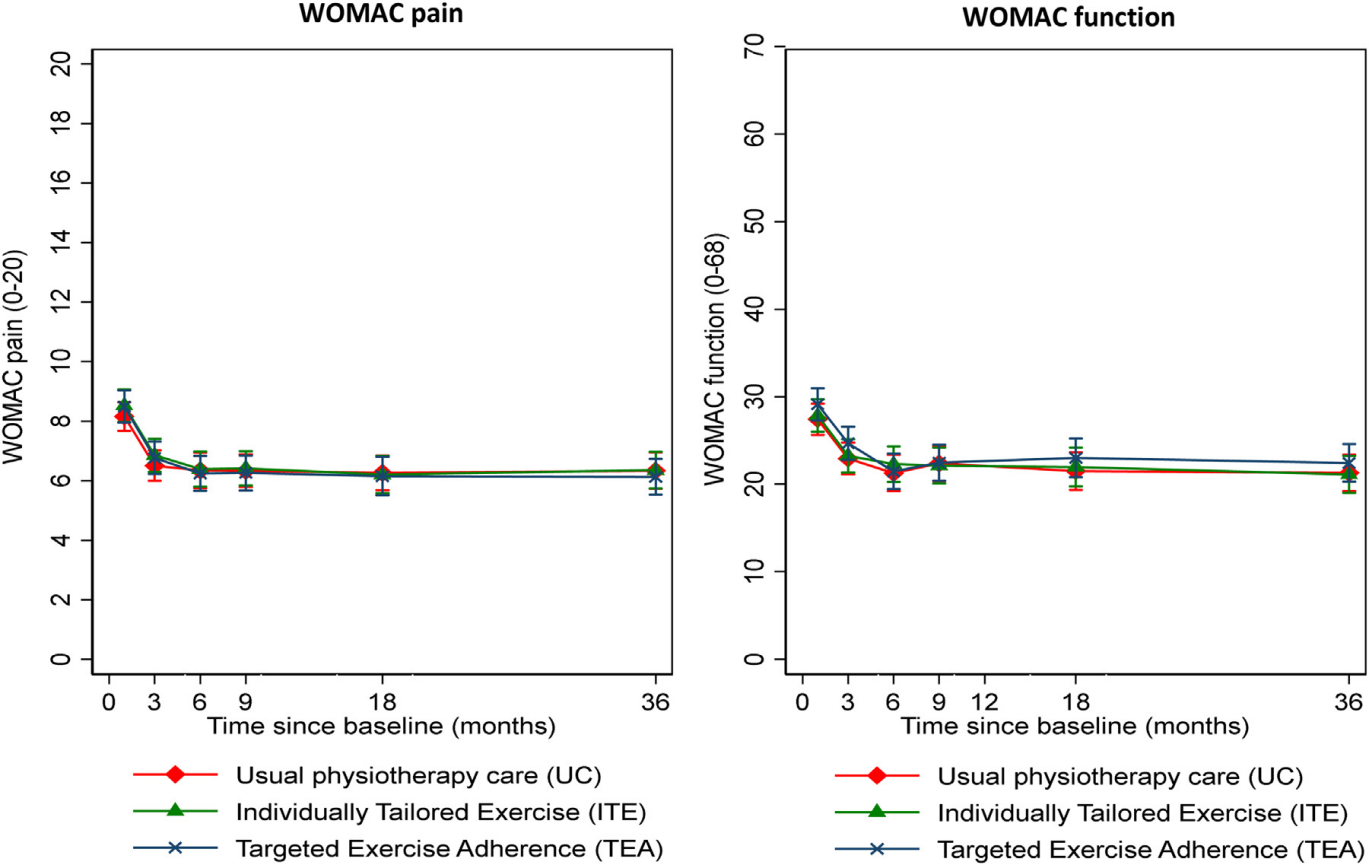
Means and 95% confidence intervals for the primary outcome at each time point.

There were no significant differences in the change in WOMAC pain or function at 6 months between UC and either ITE or TEA (see fig 3 and table 3). Longer-term outcomes at 9, 18, and 36 months remained at similar levels to those seen at 6 months and findings were similar with adjustment for baseline imbalance, imputation of missing data, and adjustment for within-physical therapist clustering (appendix 3). The per protocol analysis also demonstrated no statistically significant differences in the change in WOMAC pain or function scores (table 3). The longitudinal analysis of the mean outcome trajectory for the WOMAC pain and function scores also did not show any significant differences in the mean trajectory by intervention group. There were within group improvements in pain and function in all 3 groups, with most improvement occurring in the first 3 months, but no significant differences between the groups at any time-point.

**Table 3:** Summary of results for the primary outcomes at each time-point

Outcome Measure	3 Months	6 Months	9 Months	18 Months	36 Months
Imputed data: N	514	514	514	514	514
WOMAC pain (0-20)					
UC: Mean (SD)	6.5 (3.4)	6.3 (4.0)	6.3 (3.7)	6.3 (3.9)	6.3 (4.1)
ITE: Mean (SD)	6.9 (3.7)	6.4 (4.0)	6.4 (3.9)	6.2 (4.2)	6.4 (4.2)
TEA: Mean (SD)	6.8 (3.5)	6.2 (3.8)	6.3 (3.8)	6.2 (4.2)	6.1 (3.9)
UC vs ITE: Adjusted mean difference (95% CI)	0.1 (-0.6 to 0.8)	-0.3 (-1.0 to 0.4)	-0.2 (-1.0 to 0.6)	-0.3 (-1.1 to 0.5)	-0.2 (-1.1 to 0.7)
UC vs TEA: Adjusted mean difference (95% CI)	0.1 (-0.6 to 0.8)	-0.3 (-1.0 to 0.4)	-0.3 (-1.0 to 0.5)	-0.3 (-1.2 to 0.5)	-0.4 (-1.2 to 0.5)
WOMAC function (0-68)					
UC: Mean (SD)	22.9 (12.3)	21.3 (14.0)	22.4 (13.4)	21.5 (14.4)	21.3 (13.9)
ITE: Mean (SD)	23.2 (12.8)	22.3 (13.8)	22.1 (13.8)	22.0 (14.9)	21.1 (14.3)
TEA: Mean (SD)	24.7 (12.3)	21.5 (13.2)	22.5 (13.3)	23.0 (14.3)	22.4 (14.0)
UC vs ITE: Adjusted mean difference (95% CI)	-0.1 (-2.3 to 2.2)	0.5 (-1.9 to 2.9)	-0.7 (-3.2 to 1.8)	-0.2 (-2.9 to 2.6)	-0.6 (-3.6 to 2.3)
UC vs TEA: Adjusted mean difference (95% CI)	0.6 (-1.7 to 2.9)	-0.9 (-3.3 to 1.5)	-0.9 (-3.5 to 1.6)	0.4 (-2.4 to 3.1)	0.3 (-2.7 to 3.2)
Per-protocol analysis (imputed data):					
WOMAC pain (0-20)					
UC: N, Mean (SD)	156, 6.4 (3.5)	156, 6.2 (4.1)	156, 6.3 (3.8)	156, 6.2 (4.0)	156, 6.1 (4.1)
ITE: N, Mean (SD)	109, 6.8 (3.6)	109, 6.4 (4.0)	109, 6.5 (4.0)	109, 6.2 (4.1)	109, 6.5 (4.2)
TEA: N, Mean (SD)	78, 6.2 (2.8)	78, 5.8 (3.4)	78, 5.9 (3.6)	78, 5.4 (3.7)	78, 5.9 (3.7)
UC vs ITE: Adjusted mean difference (95% CI)	0.1 (-0.6 to 0.8)	-0.2 (-1.0 to 0.6)	-0.1 (-0.9 to 0.7)	-0.2 (-1.1 to 0.7)	0.3 (-0.7 to 1.3)
UC vs TEA: Adjusted mean difference (95% CI)	-0.3 (-1.1 to 0.5)	-0.6 (-1.5 to 0.3)	-0.5 (-1.4 to 0.4)	-0.8 (-1.8 to 0.2)	-0.1 (-1.2 to 1.0)
WOMAC function (0-68)					
UC: N, Mean (SD)	156, 22.4 (12.3)	156, 20.4 (14.1)	156, 22.1 (13.6)	156, 21.0 (14.3)	156, 20.5 (13.8)
ITE: N, Mean (SD)	109, 22.6 (12.6)	109, 21.7 (13.8)	109, 22.0 (13.7)	109, 21.5 (14.4)	109, 21.6 (14.4)
TEA: N, Mean (SD)	78, 22.4 (10.0)	78, 20.4 (11.8)	78, 20.8 (12.6)	78, 20.5 (12.6)	78, 21.5 (13.3)
UC vs ITE: Adjusted mean difference (95% CI)	-0.4 (-2.8 to 1.9)	0.1 (-2.6 to 2.9)	-0.9 (-3.7 to 2.0)	-0.4 (-3.3 to 2.6)	0.6 (-2.6 to 3.9)
UC vs TEA: Adjusted mean difference (95% CI)	-1.2 (-3.9 to 1.4)	-1.4 (-4.5 to 1.6)	-2.4 (-5.6 to 0.8)	-1.5 (-4.8 to 1.9)	0.4 (-3.2 to 4.1)

NOTE. Figures are presented after imputation of missing data and after adjustment for baseline WOMAC scores, age, sex, onset of knee problem, and treatment center unless otherwise stated. Higher WOMAC scores=worse outcome.

Abbreviation: CI, confidence interval.

Analyses of secondary outcomes showed consistent results overall, of no statistically significant differences in the change in outcomes between UC and either ITE or TEA (table 4), albeit the UC and TEA comparison for the PASE score at 9 months is marginally significant. Participants in all 3 groups, on average, reported that they felt they had greater control over their knee problem and were less concerned about their knee problem compared with baseline. At 6 months, the proportion of participants in all groups meeting the OMERACT-OARSI responder criteria was around 50% and remained relatively stable over the longer-term follow-ups.

**Table 4:** Summary of results for all secondary outcome measures

Outcome Measure	3 Months N=514	6 Months N=514	9 Months N=514	18 Months N=514	36 Months N=514
OARSI responder criteria*					
UC: N (%)	79 (45)	88 (50)	79 (45)	82 (47)	88 (50)
ITE: N (%)	81 (46)	90 (51)	86 (49)	88 (50)	84 (48)
TEA: N (%)	73 (45)	90 (55)	83 (51)	83 (51)	80 (49)
UC vs ITE: Adjusted odds ratio (95% CI)	1.1 (0.7 to 1.7)	1.1 (0.7 to 1.8)	1.3 (0.8 to 2.1)	1.1 (0.7 to 1.9)	0.9 (0.6 to 1.5)
UC vs TEA: Adjusted odds ratio (95% CI)	1.0 (0.6 to 1.7)	1.3 (0.8 to 2.0)	1.3 (0.8 to 2.1)	1.2 (0.7 to 1.9)	0.9 (0.6 to 1.5)
WOMAC stiffness (0-8)					
UC: Mean (SD)	3.1 (1.8)	2.9 (1.8)	3.0 (1.8)	2.8 (1.8)	2.9 (1.9)
ITE: Mean (SD)	3.1 (1.7)	3.1 (1.9)	3.0 (1.8)	2.9 (1.8)	2.9 (1.9)
TEA: Mean (SD)	3.1 (1.7)	2.9 (1.7)	3.1 (1.7)	3.1 (1.9)	2.8 (1.8)
UC vs ITE: Adjusted mean difference (95% CI)	0.0 (-0.3 to 0.4)	0.2 (-0.2 to 0.5)	0.0 (-0.4 to 0.3)	0.1 (-0.3 to 0.4)	0.0 (-0.4 to 0.5)
UC vs TEA: Adjusted mean difference (95% CI)	0.0 (-0.4 to 0.3)	-0.0 (-0.4 to 0.3)	0.0 (-0.3 to 0.4)	0.2 (-0.1 to 0.6)	-0.1 (-0.5 to 0.3)
PASE (0-590)					
UC: Mean (SD)	198 (87)	186 (85)	168 (76)	176 (89)	169 (87)
ITE: Mean (SD)	187 (85)	188 (87)	160 (84)	173 (79)	172 (87)
TEA: Mean (SD)	192 (93)	195 (95)	186 (87)	170 (76)	171 (84)
UC vs ITE: Adjusted mean difference (95% CI)	-8.4 (-27.4 to 10.6)	4.3 (-15.9 to 24.6)	-4.9 (-24.9 to 15.1)	-1.8 (-20.6 to 17.0)	3.8 (-15.3 to 22.8)
UC vs TEA: Adjusted mean difference (95% CI)	-5.5 (-24.4 to 13.4)	9.3 (-9.7 to 28.3)	19.0 (0.8 to 37.1)	-5.4 (-25.6 to 14.8)	1.8 (-18.2 to 21.8)
Body-mass index (kg/m^2^)					
UC: Mean (SD)	29.2 (5.7)	29.2 (5.6)	29.2 (5.7)	28.9 (5.4)	28.8 (5.4)
ITE: Mean (SD)	29.3 (5.6)	29.1 (5.8)	29.3 (5.8)	28.8 (5.6)	28.9 (5.5)
TEA: Mean (SD)	30.0 (5.5)	29.8 (5.5)	29.7 (5.5)	29.7 (5.4)	29.6 (5.3)
UC vs ITE: Adjusted mean difference (95% CI)	0.1 (-0.2 to 0.4)	-0.1 (-0.5 to 0.3)	0.1 (-0.3 to 0.6)	-0.1 (-0.6 to 0.4)	0.1 (-0.5 to 0.7)
UC vs TEA: Adjusted mean difference (95% CI)	0.3 (0.0 to 0.5)	0.0 (-0.3 to 0.4)	0.0 (-0.5 to 0.5)	0.3 (-0.2 to 0.8)	0.4 (-0.2 to 1.0)
Used local facilities or opportunities that involved any form of physical activity in the last 7 days					
UC: N (%)	100 (57)	89 (51)	72 (41)	82 (47)	89 (51)
ITE: N (%)	95 (54)	76 (43)	65 (37)	90 (51)	74 (42)
TEA: N (%)	90 (55)	80 (49)	82 (50)	72 (44)	72 (44)
UC vs ITE: Adjusted odds ratio (95% CI)	0.9 (0.5 to 1.6)	0.8 (0.5 to 1.3)	0.9 (0.5 to 1.7)	1.3 (0.8 to 2.1)	0.7 (0.4 to 1.3)
UC vs TEA: Adjusted odds ratio (95% CI)	1.1 (0.6 to 1.9)	1.1 (0.7 to 1.8)	1.9 (1.1 to 3.3)	1.0 (0.6 to 1.7)	0.8 (0.5 to 1.4)
Generalized Anxiety Disorder Assessment (GAD-7) (0-21)					
UC: Median (IQR)	0.8 (0.0, 3.2)	1.0 (0.0, 4.0)	†	1.0 (0.0, 4.1)	0.8 (0.0, 4.0)
ITE: Median (IQR)	1.0 (0.0, 3.9)	1.0 (0.0, 3.5)	†	1.1 (0.0, 4.5)	0.8 (0.0, 3.9)
TEA: Median (IQR)	1.1 (0.0, 4.6)	1.0 (0.0, 3.9)	†	0.4 (0.0, 3.8)	0.9 (0.0, 4.1)
UC vs ITE: Adjusted mean‡ difference (95% CI)	0.2 (-0.5 to 0.9)	-0.3 (-1.0 to 0.4)	†	0.4 (-0.4 to 1.2)	0.3 (-0.5 to 1.1)
UC vs TEA: Adjusted mean‡ difference (95% CI)	0.5 (-0.2 to 1.2)	0.2 (-0.6 to 0.9)	†	-0.1 (-0.8 to 0.7)	0.5 (-0.3 to 1.4)
Participant Health Questionnaire Depressive Scale (PHQ-8) (0-24)					
UC: Median (IQR)	2.0 (0.1, 5.0)	2.0 (0.0, 4.0)	†	2.0 (0.0, 5.9)	2.1 (0.0, 5.8)
ITE: Median (IQR)	2.0 (0.0, 5.0)	2.0 (0.0, 4.4)	†	2.4 (0.1, 6.0)	2.0 (0.0, 5.7)
TEA: Median (IQR)	2.0 (0.0, 5.0)	1.6 (0.0, 4.2)	†	2.0 (0.0, 5.2)	1.9 (0.0, 5.1)
UC vs ITE: Adjusted mean‡ difference (95% CI)	0.4 (-0.4 to 1.2)	0.3 (-0.4 to 1.1)	†	0.5 (-0.3 to 1.4)	-0.1 (-1.1 to 0.8)
UC vs TEA: Adjusted mean‡ difference (95% CI)	0.6 (-0.2 to 1.3)	0.5 (-0.3 to 1.3)	†	0.3 (-0.6 to 1.2)	0.1 (-0.9 to 1.1)
Self-Efficacy for Exercise Scale (SEE) (0-10)					
UC: Mean (SD)	5.7 (2.1)	5.4 (2.3)	†	†	†
ITE: Mean (SD)	5.9 (2.3)	5.8 (2.1)	†	†	†
TEA: Mean (SD)	5.5 (2.4)	5.7 (2.2)	†	†	†
UC vs ITE: Adjusted mean difference (95% CI)	0.2 (-0.2 to 0.7)	0.4 (-0.1 to 0.9)	†	†	†
UC vs TEA: Adjusted mean difference (95% CI)	-0.1 (-0.6 to 0.3)	0.4 (-0.1 to 0.9)	†	†	†
Outcome Expectations for Exercise Scale (OEE)—positive subscale (1-5)					
UC: Mean (SD)	4.0 (0.6)	3.9 (0.6)	†	†	†
ITE: Mean (SD)	4.0 (0.6)	4.0 (0.6)	†	†	†
TEA: Mean (SD)	3.9 (0.5)	3.9 (0.6)	†	†	†
UC vs ITE: Adjusted mean difference (95% CI)	0.0 (-0.1 to 0.1)	0.0 (-0.1 to 0.2)	†	†	†
UC vs TEA: Adjusted mean difference (95% CI)	-0.1 (-0.2 to 0.1)	0.0 (-0.1 to 0.1)	†	†	†
Outcome Expectations for Exercise Scale (OEE)—negative subscale (1-5)§					
UC: Mean (SD)	3.8 (0.7)	3.8 (0.8)	†	†	†
ITE: Mean (SD)	3.9 (0.8)	3.9 (0.9)	†	†	†
TEA: Mean (SD)	3.7 (0.8)	3.8 (0.8)	†	†	†
UC vs ITE: Adjusted mean difference (95% CI)	0.1 (-0.1 to 0.2)	0.1 (0.0 to 0.3)	†	†	†
UC vs TEA: Adjusted mean difference (95% CI)	0.0 (-0.2 to 0.1)	0.2 (0.0 to 0.3)	†	†	†
How much does your knee pain affect your life? (0-10)					
UC: Mean (SD)	4.5 (2.3)	4.3 (2.5)	†	†	†
ITE: Mean (SD)	4.4 (2.3)	4.2 (2.5)	†	†	†
TEA: Mean (SD)	4.6 (2.4)	4.3 (2.5)	†	†	†
UC vs ITE: Adjusted mean difference (95% CI)	-0.1 (-0.6 to 0.4)	-0.1 (-0.6 to 0.4)	†	†	†
UC vs TEA: Adjusted mean difference (95% CI)	0.1 (-0.3 to 0.6)	0.0 (-0.5 to 0.5)	†	†	†
How long do you think your knee pain will continue? (0-10)					
UC: Median (IQR)	8.0 (5.1, 10.0)	8.8 (5.7, 10.0)	†	†	†
ITE: Median (IQR)	8.0 (5.0, 10.0)	8.0 (5.3, 10.0)	†	†	†
TEA: Median (IQR)	7.8 (5.0, 10.0)	8.0 (5.0, 10.0)	†	†	†
UC vs ITE: Adjusted mean‡ difference (95% CI)	-0.2 (-0.8 to 0.4)	-0.1 (-0.7 to 0.6)	†	†	†
UC vs TEA: Adjusted mean‡ difference (95% CI)	-0.2 (-0.8 to 0.4)	-0.2 (-0.8 to 0.5)	†	†	†
How much control do you feel you have over your knee pain? (0-10)					
UC: Mean (SD)	5.0 (2.6)	5.0 (2.5)	†	†	†
ITE: Mean (SD)	5.2 (2.6)	5.4 (2.6)	†	†	†
TEA: Mean (SD)	4.6 (2.6)	5.3 (2.6)	†	†	†
UC vs ITE: Adjusted mean difference (95% CI)	0.3 (-0.3 to 0.9)	0.5 (-0.1 to 1.1)	†	†	†
UC vs TEA: Adjusted mean difference (95% CI)	-0.3 (-0.9 to 0.3)	0.4 (-0.2 to 1.0)	†	†	†
How much do you think treatment can help your knee pain? (0-10)					
UC: Median (IQR)	6.2 (5.0, 8.0)	5.9 (4.3, 8.0)	†	†	†
ITE: Median (IQR)	7.0 (5.0, 8.0)	6.9 (4.3, 8.0)	†	†	†
TEA: Median (IQR)	7.0 (5.0, 8.0)	7.0 (5.0, 9.0)	†	†	†
UC vs ITE: Adjusted mean‡ difference (95% CI)	0.2 (-0.4 to 0.8)	0.3 (-0.3 to 0.9)	†	†	†
UC vs TEA: Adjusted mean‡ difference (95% CI)	0.2 (-0.4 to 0.7)	0.8 (0.2 to 1.4)	†	†	†
How much do you experience symptoms from your knee pain? (0-10)					
UC: Mean (SD)	5.3 (2.3)	5.0 (2.3)	†	†	†
ITE: Mean (SD)	5.2 (2.3)	4.9 (2.5)	†	†	†
TEA: Mean (SD)	5.4 (2.3)	5.0 (2.3)	†	†	†
UC vs ITE: Adjusted mean difference (95% CI)	-0.1 (-0.5 to 0.4)	-0.1 (-0.6 to 0.4)	†	†	†
UC vs TEA: Adjusted mean difference (95% CI)	0.2 (-0.3 to 0.7)	0.0 (-0.5 to 0.5)	†	†	†
How concerned are you about your knee pain? (0-10)					
UC: Median (IQR)	6.6 (5.0, 8.0)	5.3 (3.0, 8.0)	†	†	†
ITE: Median (IQR)	6.1 (4.0, 8.4)	5.7 (3.0, 8.0)	†	†	†
TEA: Median (IQR)	6.4 (5.0, 8.2)	5.1 (3.0, 8.0)	†	†	†
UC vs ITE: Adjusted mean‡ difference (95% CI)	0.0 (-0.5 to 0.5)	0.2 (-0.4 to 0.8)	†	†	†
UC vs TEA: Adjusted mean‡ difference (95% CI)	0.1 (-0.4 to 0.7)	0.0 (-0.6 to 0.7)	†	†	†
How well do you feel you understand your knee pain? (0-10)					
UC: Mean (SD)	7.0 (2.5)	7.2 (2.4)	†	†	†
ITE: Mean (SD)	7.2 (2.5)	7.6 (2.5)	†	†	†
TEA: Mean (SD)	6.9 (2.6)	7.5 (2.5)	†	†	†
UC vs ITE: Adjusted mean difference (95% CI)	0.2 (-0.3 to 0.7)	0.4 (-0.1 to 1.0)	†	†	†
UC vs TEA: Adjusted mean difference (95% CI)	0.0 (-0.5 to 0.6)	0.5 (-0.1 to 1.1)	†	†	†
How much does your knee pain affect you emotionally? (0-10)					
UC: Mean (SD)	4.1 (2.8)	3.4 (2.9)	†	†	†
ITE: Mean (SD)	3.9 (2.9)	3.6 (2.9)	†	†	†
TEA: Mean (SD)	4.2 (2.8)	3.6 (2.9)	†	†	†
UC vs ITE: Adjusted mean difference (95% CI)	-0.3 (-0.8 to 0.3)	0.2 (-0.4 to 0.8)	†	†	†
UC vs TEA: Adjusted mean difference (95% CI)	0.0 (-0.5 to 0.6)	0.2 (-0.4 to 0.8)	†	†	†
Accelerometer Data|| (Sub-sample of Trial Participants)	N=50	N=47	N=51	N=48	N=40
Average¶ number of counts per minute (measured by accelerometers)					
UC: N: Median (IQR)	15, 346 (124, 460)	14, 338 (138, 424)	15, 306 (176, 414)	14, 222 (117, 440)	14, 211 (126, 366)
ITE: N: Median (IQR)	19, 257 (170, 278)	17, 209 (159, 290)	20, 201 (157, 316)	18, 238 (174, 348)	17, 216 (197, 236)
TEA: N: Median (IQR)	16, 239 (193, 324)	16, 264 (205, 278)	16, 223 (166, 250)	16, 205 (161, 283)	14, 177 (145, 208)
UC vs ITE: Adjusted# mean‡ difference (95% CI)	-28 (-84 to 28)	-66 (-147 to 15)	-47 (-101 to 7)	-17 (-86 to 52)	-7 (-73 to 60)
UC vs TEA: Adjusted# mean‡ difference (95% CI)	-51 (-108 to 7)	-53 (-138 to 31)	-61 (-117 to -4)	-56 (-130 to 19)	-48 (-119 to 22)
Proportion meeting physical activity guidelines (measured by accelerometers**)					
UC: N (%)	5 (33)	4 (29)	5 (33)	3 (21)	1 (7)
ITE: N (%)	0 (0)	0 (0)	0 (0)	3 (17)	2 (12)
TEA: N (%)	3 (19)	1 (6)	0 (0)	1 (6)	1 (7)
UC vs ITE: Adjusted# odds ratio (95% CI)	††	††	††	††	††
UC vs TEA: Adjusted# odds ratio (95% CI)	††	††	††	††	††

NOTE. Figures are presented after imputation of missing data (with the exception of data from the accelerometers) and after adjustment for the baseline score on the outcome of interest (with the exception of the OARSI responder criteria), age, sex, onset of knee problem, and treatment center, unless otherwise stated. WOMAC stiffness higher score=more severe stiffness; PASE higher score=more active; SEE higher score=more confident that exercise can be done; OEE positive and negative subscales higher score=higher expectations that exercise will be beneficial; GAD-7 higher score=more anxious; PHQ-8 higher score=more depressed; IPQR—affects life, higher score=more affected; IPQR—duration, higher score=lasts a longer time; IPQR—personal control, higher score=more control; IPQR—treatment control, higher score=higher belief treatment can control; IPQR—symptom experience, higher score=more symptoms that are more severe; IPQR—concern, higher score=more concerned; IPQR—understanding, higher score=more understanding; IPQR—emotion, higher score=more emotionally affected.

Abbreviations: CI, confidence interval; IQR, inter-quartile range; SD, standard deviation.

⁎ Participants met the OARSI responder criteria if (a) relative change in WOMAC pain or function was ≥50% and absolute change was ≥20 or (b) at least 2 of the following applied: relative change in pain ≥20% and absolute change ≥10, relative change in function ≥20% and absolute change ≥10 or participants reported they were improved, much improved, or completely recovered on the global assessment of change question. Absolute change (baseline—follow-up score) and relative change (absolute change/baseline score) were calculated after WOMAC measures were scaled from 1 to 101 to avoid dividing by 0 when calculating relative change.^26^

† Data not collected at this time point.

‡ Mean differences are presented, despite a skewed distribution for the outcome at the absolute time point, as, when adjusted for the baseline value of interest, model residuals followed a normal distribution.

§ Reverse scored, that is, a higher score on the negative subscale indicates higher expectations of the benefit of exercise.

|| Participants are only included if they have worn the monitor for at least 5 days for 10 hours or more. Valid time is calculated assuming that any consecutive runs of zero count lasting for 60 minutes or more are counted as non-wear.

¶ An average score was calculated for each participant by averaging the total number of counts across the valid time for which the accelerometer was worn.

# Model adjusted for baseline only (adjusting for all a priori model covariates gave unstable model results due to the small sample size used for the analysis).

⁎⁎ Defined as participants completing 150 minutes each week of moderate intensity physical activity (accumulated in bouts of 10 minutes or more) or 75 minutes of vigorous intensity activity spread across the week (adapted from Ref. 39). Bouts calculated using a drop-time of 2 minutes.^40^ Missing days of data are imputed using the average of the average count for days where data are present.

†† Not calculated due to small N.

Overall, perceived levels of treatment acceptability and credibility were high in all groups and remained so even at 36 months follow-up (table 5). At 3 months, exercise adherence was high in all groups, with 75% or more agreeing or strongly agreeing that they had completed their exercises as often as they had been advised. In the UC group this reduced at 6 months, and then fell below 50% at longer-term follow-ups. Self-reported exercise adherence was maintained at higher levels for longer in the ITE and TEA groups, but differences between groups at the longer term follow-ups (18 and 36 months) were small. Results for the primary outcome at the primary endpoint also did not differ depending on whether the participant reported high or low levels of exercise adherence (appendix 4).

**Table 5:** Treatment credibility and exercise adherence at each follow-up time-point

	3 Months			6 Months			9 Months			18 Months			36 Months		
Question	UC N=143	ITE N=146	TEA N=136	UC N=142	ITE N=140	TEAN=131	UC N=139	ITE N=135	TEA N=129	UC N=141	ITE N=134	TEA N=120	UC N=122	ITE N=125	TEA N=115
Confident that treatment received can help knee problem															
Very confident	30 (21)	49 (34)	45 (33)	26 (19)	43 (31)	51 (40)	22 (16)	31 (23)	39 (31)	15 (11)	30 (23)	31 (27)	18 (15)	23 (19)	33 (30)
Quite confident	70 (50)	66 (46)	63 (47)	63 (45)	62 (45)	45 (35)	59 (44)	66 (49)	53 (42)	59 (44)	59 (44)	49 (42)	45 (38)	52 (42)	42 (38)
Neither	13 (9)	9 (6)	11 (8)	15 (11)	8 (6)	13 (10)	23 (17)	13 (10)	9 (7)	28 (21)	15 (11)	13 (11)	19 (16)	19 (15)	11 (10)
Not very confident	22 (16)	16 (11)	14 (10)	26 (19)	19 (14)	10 (8)	24 (18)	17 (13)	18 (14)	24 (18)	19 (14)	15 (13)	26 (22)	19 (15)	20 (18)
Not at all confident	5 (4)	5 (3)	2 (1)	9 (6)	6 (4)	10 (8)	6 (4)	7 (5)	7 (6)	9 (7)	10 (8)	9 (8)	9 (8)	10 (8)	5 (5)
Confident in recommending this treatment to a friend															
Very confident	33 (24)	57 (39)	50 (37)	32 (23)	59 (43)	50 (39)	29 (21)	48 (36)	46 (37)	26 (19)	39 (29)	41 (35)	22 (19)	28 (23)	45 (41)
Quite confident	70 (50)	59 (41)	60 (45)	63 (45)	47 (34)	51 (40)	58 (43)	57 (43)	50 (40)	61 (45)	54 (41)	45 (38)	52 (44)	61 (50)	34 (31)
Neither	16 (11)	13 (9)	13 (10)	22 (16)	21 (15)	9 (7)	31 (23)	14 (10)	10 (8)	26 (19)	13 (10)	15 (13)	17 (14)	15 (12)	15 (14)
Not very confident	19 (14)	10 (7)	8 (6)	17 (12)	7 (5)	12 (9)	10 (7)	10 (7)	16 (13)	18 (13)	15 (11)	10 (9)	19 (16)	12 (10)	12 (11)
Not at all confident	2 (1)	6 (4)	3 (2)	6 (4)	4 (3)	7 (5)	7 (5)	5 (4)	4 (3)	4 (3)	12 (9)	6 (5)	8 (7)	6 (5)	4 (4)
Treatment makes sense to you															
Very logical	46 (33)	73 (50)	57 (42)	38 (27)	70 (51)	61 (47)	39 (29)	51 (38)	48 (38)	36 (27)	45 (34)	40 (34)	38 (32)	35 (28)	40 (36)
Quite logical	76 (54)	61 (42)	68 (50)	78 (56)	50 (36)	54 (42)	70 (52)	65 (49)	54 (43)	64 (47)	65 (49)	51 (44)	48 (41)	64 (52)	47 (43)
No opinion	12 (9)	3 (2)	6 (4)	17 (12)	9 (7)	6 (5)	21 (16)	8 (6)	11 (9)	26 (19)	14 (11)	14 (12)	18 (15)	16 (13)	14 (13)
Not very logical	5 (4)	6 (4)	3 (2)	7 (5)	7 (5)	5 (4)	5 (4)	7 (5)	11 (9)	8 (6)	2 (2)	3 (3)	10 (8)	5 (4)	8 (7)
Not at all logical	1 (1)	2 (1)	1 (1)	0 (0)	2 (1)	3 (2)	0 (0)	3 (2)	2 (2)	1 (1)	7 (5)	9 (8)	4 (3)	3 (2)	1 (1)
Treatment would be successful in helping other types of problems															
Very successful	23 (16)	22 (15)	30 (22)	18 (13)	27 (20)	39 (30)	24 (18)	23 (17)	30 (24)	21 (16)	26 (20)	23 (20)	15 (13)	18 (15)	27 (25)
Quite successful	55 (39)	73 (50)	62 (46)	65 (46)	60 (43)	43 (33)	51 (38)	71 (53)	43 (34)	52 (39)	56 (42)	50 (43)	44 (38)	50 (41)	42 (38)
No opinion	54 (39)	46 (32)	41 (30)	54 (39)	46 (33)	40 (31)	53 (39)	34 (25)	46 (36)	52 (39)	43 (33)	35 (30)	46 (40)	50 (41)	34 (31)
Not very successful	7 (5)	2 (1)	1 (1)	3 (2)	4 (3)	6 (5)	6 (4)	3 (2)	7 (6)	9 (7)	5 (4)	4 (3)	8 (7)	4 (3)	6 (5)
Not at all successful	1 (1)	2 (1)	1 (1)	0 (0)	1 (1)	1 (1)	1 (1)	3 (2)	1 (1)	1 (1)	2 (2)	5 (4)	3 (3)	1 (1)	1 (1)
Total treatment credibility score (0-16)*: Median (interquartile range)	12 (9, 14)	13 (11, 15)	12 (11, 15)	12 (9, 13)	13 (10, 14)	12 (10, 15)	11 (8, 13)	12 (10, 14)	12 (10.15)	11 (8.13)	12 (9.14)	12 (9.14)	11 (8.13)	12 (9.13)	12 (9.15)
Been doing exercises as often as advised															
Strongly agree	23 (16)	39 (27)	39 (29)	12 (9)	27 (20)	36 (28)	15 (11)	23 (17)	22 (17)	12 (9)	19 (14)	18 (15)	6 (5)	9 (7)	15 (13)
Agree	82 (59)	78 (54)	71 (52)	64 (46)	66 (48)	63 (49)	48 (36)	52 (39)	72 (57)	50 (37)	42 (32)	42 (36)	38 (32)	47 (39)	48 (43)
Not sure	13 (9)	12 (8)	12 (9)	21 (15)	21 (15)	11 (9)	23 (17)	12 (9)	14 (11)	17 (13)	15 (11)	22 (19)	20 (17)	11 (9)	16 (14)
Disagree	20 (14)	13 (9)	12 (9)	34 (24)	20 (15)	17 (13)	36 (27)	39 (29)	17 (13)	42 (31)	44 (33)	25 (21)	38 (32)	41 (34)	28 (25)
Strongly disagree	2 (1)	3 (2)	2 (1)	9 (6)	3 (2)	1 (1)	11 (8)	7 (5)	2 (2)	13 (10)	12 (9)	10 (9)	16 (14)	13 (11)	5 (4)
Exercise frequency in the last month															
Never	6 (4)	4 (3)	2 (2)	18 (13)	11 (8)	3 (2)	21 (16)	20 (15)	16 (13)	36 (26)	34 (26)	30 (26)	41 (35)	40 (33)	28 (25)
Once a week	5 (4)	4 (3)	1 (1)	11 (8)	3 (2)	3 (2)	17 (13)	20 (15)	5 (4)	21 (15)	21 (16)	9 (8)	16 (14)	14 (11)	12 (11)
Twice a week	11 (8)	3 (2)	2 (2)	23 (17)	18 (13)	10 (8)	24 (18)	18 (14)	7 (6)	23 (17)	23 (18)	13 (11)	11 (9)	17 (14)	7 (6)
Three times a week	11 (8)	17 (12)	11 (8)	12 (9)	23 (17)	19 (15)	13 (10)	18 (14)	23 (18)	17 (13)	15 (11)	12 (10)	12 (10)	20 (16)	16 (14)
Four times a week	17 (12)	16 (11)	7 (5)	14 (10)	17 (12)	14 (11)	13 (10)	11 (8)	13 (10)	5 (4)	3 (2)	8 (7)	8 (7)	4 (3)	11 (10)
Five times a week	21 (15)	8 (6)	11 (8)	19 (14)	10 (7)	10 (8)	11 (8)	5 (4)	11 (9)	8 (6)	11 (8)	13 (11)	3 (3)	5 (4)	10 (9)
Six times a week	3 (2)	10 (7)	10 (8)	10 (7)	10 (7)	6 (5)	3 (2)	3 (2)	7 (6)	1 (1)	1 (1)	3 (3)	1 (1)	3 (2)	3 (3)
Once every day	47 (34)	54 (38)	43 (33)	22 (16)	32 (23)	30 (24)	26 (20)	34 (26)	30 (24)	20 (15)	19 (15)	21 (18)	18 (16)	19 (15)	18 (16)
Twice every day	19 (14)	27 (19)	44 (34)	8 (6)	13 (9)	31 (25)	4 (3)	2 (2)	15 (12)	5 (4)	4 (3)	6 (5)	6 (5)	1 (1)	7 (6)
Exercise duration															
Less than 5 minutes	9 (6)	7 (5)	2 (2)	9 (7)	7 (5)	7 (5)	17 (13)	5 (4)	12 (10)	8 (6)	15 (12)	9 (8)	8 (7)	13 (11)	8 (8)
5 minutes to <10 minutes	23 (17)	27 (19)	36 (27)	28 (21)	28 (20)	34 (27)	25 (20)	41 (32)	30 (24)	28 (22)	44 (34)	28 (25)	26 (23)	26 (22)	22 (21)
10 minutes to <15 minutes	33 (24)	42 (29)	43 (32)	36 (26)	44 (32)	38 (30)	32 (25)	34 (27)	40 (32)	42 (33)	25 (19)	28 (25)	24 (22)	33 (28)	29 (27)
15 minutes to <1 hour	62 (45)	58 (40)	49 (37)	50 (37)	48 (35)	44 (34)	39 (30)	37 (29)	33 (27)	23 (18)	22 (17)	27 (24)	26 (23)	18 (16)	28 (26)
An hour or more	8 (6)	8 (6)	2 (2)	3 (2)	2 (1)	3 (2)	1 (1)	1 (1)	3 (2)	4 (3)	3 (2)	3 (3)	3 (3)	1 (1)	2 (2)
I don't do the exercises	4 (3)	2 (1)	1 (1)	10 (7)	9 (7)	2 (2)	14 (11)	10 (8)	6 (5)	23 (18)	20 (16)	16 (14)	24 (22)	25 (22)	17 (16)

NOTE. Figures are N (%), unless otherwise stated. Analysis completed on non-imputed data as only the primary and secondary outcomes (excluding accelerometer data) were included in the imputation model.

⁎ Total treatment credibility score is calculated as the sum of the 4 treatment credibility questions when each question is coded on a 0-4 scale; higher score=treatment more credible.

Physical activity, measured using the PASE, and via accelerometry within the subsample (that met the criteria for valid wear time), showed similar small increases in all 3 groups at 6 months but returned to baseline (or below baseline) levels at 36 months. Self-reported use of physical activity facilities in the last 7 days increased in all 3 groups from baseline to 6 months and stayed higher even at longer-term follow-up.

## Discussion

The key findings from this large, pragmatic trial are that there were no significant differences between the intervention groups. Results of secondary outcomes and sensitivity analyses, including a treated-per-protocol analysis, support the same conclusion. Thus, there is no evidence that our approaches to increasing individual tailoring of, and targeting adherence to, exercise for adults with knee OA, are more effective than UC for improving pain and physical function. All 3 groups showed improvements in pain and function in line with those reported in previous meta-analyses,^2^ and approximately half of participants in all 3 treatment groups were classified as treatment responders.

The lack of significant differences between groups adds to the debate about the mechanisms of effect of exercise for knee OA.^34^, ^35^, ^36^ It questions the assumptions that “doing more” lower limb exercise, with greater individualization, exercise progression, and supervision leads to better pain and function. It also questions the assumption that supporting patients to identify and engage with general aerobic physical activities they enjoy leads to greater adherence to exercise and activity, and thus greater improvements in pain and function. The good outcomes, on average, achieved in the usual care intervention group and indeed by all 3 groups suggest that other factors may be important. Our nested qualitative research in this trial highlighted potential explanations that include the value of reassurance from physical therapists that exercise was safe, the opportunity to exercise with the support of a physical therapist who could address their concerns about exercise, and the therapeutic relation they develop with the physical therapist.^13^ However, there may be several other explanations (and potential trial limitations) for the results; lack of sufficient difference between interventions, and lack of intervention fidelity particularly in TEA. Our trial was designed and delivered within the UK NHS, and thus the decision about the number of treatment contacts was influenced by what physical therapists and their managers perceived would be deliverable, given that current practice is typically an average up to 4 treatment sessions.^19^ We protocolized between 8 and 10 treatment contacts in the TEA intervention and, on average, participants received 7. This may not have been sufficient to facilitate long-term behavior change. Within TEA, overall, fidelity was low with only the “simpler” educational and behavioral tools (eg, written education materials, physical activity diaries, and pedometers) being frequently used. The range of tools within the adherence-enhancing toolkit offered considerable flexibility to the physical therapists, which may have inadvertently diluted the intended focus on increasing exercise adherence in this group.

This trial also provides no evidence that the interventions we tested lead to greater sustained changes in physical activity levels at longer-term follow-up at 18 or 36 months. While self-reported exercise adherence appeared to stay higher for longer in TEA, physical activity levels had returned to baseline levels (or below) by the longer-term follow-ups, indicating no sustained behavior change 1 year after the end of physical therapy contact. Despite this reduction in adherence, pain and function outcomes did not regress to baseline values. While this is difficult to explain, it could be related to the reassurance about exercise from physical therapists, resulting in patients being less worried about their knee OA and therefore reporting less pain and dysfunction. It might alternatively be explained by patients being recruited into the trial at a time when they are experiencing an exacerbation of symptoms. Overall, the results show that our attempts to increase individual tailoring of, and better target adherence to, exercise for adults with knee OA, were not more effective than usual exercise based care for improving pain and physical function. It is possible that different efforts are needed to sustain longer-term exercise and physical activity behavior.

### Comparison with other research

Overall, the proportion of treatment responders in each arm was similar or better, at 6 months, than reported in previous trials,^7^^,^^8^^,^^37^ and were maintained at 36 months follow-up. Interestingly, UC was associated with a higher proportion of treatment responders than in some previous trials.^7^ Our results are similar to an Australian trial comparing different exercise approaches (neuromuscular vs quadriceps muscle strengthening) for patients with knee OA.^38^ This also showed that while all groups reported improvements in pain and function over time, there were no significant differences between exercise groups. Our results build further on the subgroup analyses in a systematic review,^39^ that concluded there was uncertain evidence as to whether increased exercise time (duration, number of sessions) effects on outcomes, by showing that it does not. Our results are also in line with a recent trial comparing high intensity vs low intensity strength training vs attention control for knee OA.^40^

### Strengths and limitations

Although this trial had a large sample size, good follow-up rates over 36 months, participation of many physical therapists, and a diverse sample (eg, in terms of comorbidities), it did have potential limitations. In addition to the potential lack of sufficient difference between interventions, and intervention fidelity (particularly in TEA), we did not adjust for multiple testing in our analyses of the 2 primary outcomes and comparisons (UC vs ITE, UC vs TEA). However, given the non-significant results, this would not change our conclusions.

### Clinical and research recommendations

Although the usual exercise-based physical therapy intervention could be considered best practice, the consistent observation of decline in physical activity in all 3 groups after the end of physical therapy contact suggests that interventions that effectively increase exercise adherence need to be developed and tested. Furthermore, while the trial showed that 50% or more of participants could be classified as treatment responders, this means that up to half did not. Further research that leads to better understanding, and easier identification and prediction, of those patients who do and do not benefit from exercise, and to different types and intensities of exercise, would be useful to better target exercise treatments in future.

## Conclusions

We found no evidence that our approaches to increasing individual tailoring of, or targeting adherence to, exercise for adults with knee OA, are more effective than usual physical therapist-led care for pain and physical function in adults with knee OA. Future research needs to develop and test interventions that effectively increase exercise adherence and identify characteristics of patients with knee OA that respond to exercise.

## Figures and Tables

**Table:** 

	UC	ITE	TEA
Treatment clinic			
1	18	16	17
2	10	10	9
3	17	17	17
4	9	9	8
5	33	31	25
6	18	14	17
7	13	15	13
8	18	19	17
9	9	11	11
10	18	19	17
Mean (SD) of the number of patients treated in each clinic	16.3 (7.1)	16.1 (6.3)	15.1 (5.0)
Physiotherapist^α^			
1	**5**	0	0
2	**1**	0	0
3	**8**	0	0
4	**6**	0	0
5	**18**	0	0
6	**17**	0	0
7	**10**	0	0
8	**8**	0	0
9	**12**	0	0
10	**1**	0	0
11	**8**	0	0
12	**14**	0	0
13	**27**	0	0
14	**17**	0	0
15	**9**	0	0
16	0	**16**	0
17	0	**18**	0
18	0	**6**	0
19	0	**11**	0
20	0	**1**	0
21	0	**9**	0
22	0	**1**	0
23	0	**9**	0
24	0	**26**	0
25	0	**4**	0
26	0	**7**	0
27	0	**14**	0
28	0	**20**	0
29	0	**5**	0
30	0	**9**	0
31	0	**4**	0
32	0	0	**9**
33	0	0	**1**
34	0	0	**12**
35	0	0	**20**
36	0	0	**2**
37	0	0	**17**
38	0	0	**13**
39	1	0	**11**
40	1	0	**23**
41	0	0	**2**
42	0	1	**15**
43	0	0	**6**
44	0	0	**8**
45	0	0	**5**
46	0	0	**7**
47	0^β^	0 ^β^	0 ^β^
Mean (SD) of the number of patients treated by each physiotherapist trained to deliver the intervention	10.7 (7.0)	10.0 (7.1)	10.1 (6.7)

**Table:** 

Outcome measure	3 months	6 months	9 months	18 months	36 months
Adjusting for therapist effects (imputed data)					
WOMAC pain (0-20)					
UC vs ITE: Adjusted mean difference (95% CI)	0.1 (-0.5 to 0.7)	-0.1 (-0.8 to 0.7)	-0.1 (-0.9 to 0.7)	-0.3 (-0.9 to 0.4)	-0.1 (-0.9 to 0.6)
UC vs TEA: Adjusted mean difference (95% CI)	0.0 (-0.5 to 0.6)	-0.2 (-1.0 to 0.6)	-0.3 (-1.2 to 0.5)	-0.5 (-1.2 to 0.3)	-0.3 (-1.1 to 0.5)
WOMAC function (0-68)					
UC vs ITE: Adjusted mean difference (95% CI)	0.1 (-2.0 to 2.2)	1.1 (-0.7 to 3.0)	-0.5 (-2.8 to 1.9)	0.1 (-2.3 to 2.4)	-0.4 (-2.9 to 2.1)
UC vs TEA: Adjusted mean difference (95% CI)	0.5 (-1.5 to 2.5)	-0.6 (-2.8 to 1.7)	-1.1 (-3.5 to 1.4)	0.0 (-2.3 to 2.3)	0.5 (-1.9 to 3.0)
Removing adjusting covariates, except baseline (BL) from the model (imputed data)					
WOMAC pain (0-20)					
UC vs ITE: Mean difference adjusted for BL (95% CI)	0.1 (-0.6 to 0.8)	-0.2 (-0.9 to 0.5)	-0.1 (-0.9 to 0.7)	-0.3 (-1.1 to 0.6)	-0.2 (-1.1 to 0.7)
UC vs TEA: Mean difference adjusted for BL (95% CI)	0.1 (-0.6 to 0.8)	-0.3 (-1.0 to 0.4)	-0.3 (-1.0 to 0.5)	-0.3 (-1.2 to 0.6)	-0.4 (-1.3 to 0.5)
WOMAC function (0-68)					
UC vs ITE: Mean difference adjusted for BL (95% CI)	0.0 (-2.2 to 2.3)	0.8 (-1.7 to 3.2)	-0.5 (-3.1 to 2.1)	0.2 (-2.6 to 3.1)	-0.4 (-3.4 to 2.6)
UC vs TEA: Mean difference adjusted for BL (95% CI)	0.6 (-1.7 to 2.9)	-0.9 (-3.4 to 1.6)	-0.9 (-3.6 to 1.7)	0.5 (-2.4 to 3.3)	0.2 (-2.8 to 3.2)
Complete-case data:					
WOMAC pain (0-20)					
UC: N, Mean (SD)	142, 6.5 (3.5)	156, 6.3 (4.1)	135, 6.1 (3.7)	138, 6.3 (3.8)	124, 6.3 (4.1)
ITE: N, Mean (SD)	142, 6.7 (3.8)	151, 6.2 (4.0)	135, 6.3 (4.0)	141, 6.2 (4.3)	133, 6.3 (4.2)
TEA: N, Mean (SD)	133, 6.8 (3.6)	146, 6.3 (3.8)	124, 6.2 (3.9)	128, 6.1 (4.3)	120, 6.1 (3.9)
UC vs ITE: Adjusted mean difference (95% CI)	0.0 (-0.7 to 0.6)	-0.3 (-1.0 to 0.4)	0.0 (-0.8 to 0.7)	-0.1 (-1.0 to 0.7)	-0.1 (-1.0 to 0.8)
UC vs TEA: Adjusted mean difference (95% CI)	-0.1 (-0.8 to 0.6)	-0.4 (-1.1 to 0.3)	-0.3 (-1.1 to 0.5)	-0.3 (-1.2 to 0.6)	-0.3 (-1.2 to 0.7)
WOMAC function (0-68)					
UPC: N, Mean (SD)	140, 22.5 (12.7)	157, 21.3 (14.3)	135, 21.8 (13.4)	139, 21.2 (14.4)	118, 21.0 (14.1)
ITE: N, Mean (SD)	143, 22.5 (12.9)	151, 21.8 (13.8)	131, 21.0 (14.1)	141, 21.8 (15.1)	128, 20.5 (14.8)
TEA: N, Mean (SD)	131, 24.6 (12.8)	144, 21.3 (13.2)	124, 22.4 (13.7)	127, 22.4 (14.7)	116, 22.1 (14.2)
UC vs ITE: Adjusted mean difference (95% CI)	-0.2 (-2.4 to 2.0)	0.6 (-1.8 to 3.0)	-0.9 (-3.5 to 1.8)	0.7 (-2.1 to 3.5)	-0.1 (-3.2 to 3.0)
UC vs TEA: Adjusted mean difference (95% CI)	0.4 (-1.9 to 2.6)	-0.9 (-3.4 to 1.6)	-0.8 (-3.5 to 1.9)	0.8 (-2.1 to 3.7)	1.0 (-2.2 to 4.2)

**Table:** 

	I have been doing my exercises as often as I was advised to…..	

	Strongly agree/agree N=268	Strongly disagree/disagree N=84

WOMAC pain (0-20)		
UC: N, Mean (SD)	6.4 (4.0)	6.1 (3.8)
ITE: N, Mean (SD)	6.1 (4.2)	6.2 (3.5)
TEA: N, Mean (SD)	5.9 (3.8)	6.2 (3.7)
Interaction regression coefficient^α^ (95% CI)		
UC	0	0
ITE	0	1.0 (-0.9 to 2.9)
TEA	0	0.6 (-1.4 to 2.6)
WOMAC function (0-68)		
UC: N, Mean (SD)	22.1 (14.0)	20.0 (13.9)
ITE: N, Mean (SD)	20.9 (14.7)	20.9 (12.5)
TEA: N, Mean (SD)	20.8 (13.2)	19.6 (14.1)
Interaction regression coefficient^α^ (95% CI)		
UC	0	0
ITE	0	4.6 (-1.9 to 11.0)
TEA	0	1.5 (-5.3 to 8.3)

## Appendix 1 – Strategy to impute missing data

Multiple imputation was used to impute missing data at all time-points for outcomes shown in Appendices 6 and 7 (excluding accelerometry), and for the adjusting covariates used in the regression models. Despite not having any missing data, the variable representing the treatment effect was included in the imputation model as this variable was included in the analysis models. An imputation model was fitted using Multiple Imputation by chained equations (MICE) in STATA version 15.0 [1] and assumed that data were missing at random, as evidenced by describing the baseline characteristics of participants with and without data at each time-point. Twenty-five imputed data sets were derived to ensure the number of imputations exceeded the overall percentage of missing data [2]. Continuous outcome measures were modelled using predictive mean matching (nearest neighbours = 1[1]) and ordinal outcomes were modelled using ordinal regression. Predictive mean matching was chosen so that the imputed values remained on the same scale as their original outcome and because this method is particularly suited to modelling skewed data [2]. All ordinal outcomes were imputed using the “augment” option in STATA to avoid the problem of perfect prediction [1]. The imputation model for the global assessment of change (GAC) outcomes also included the “ascontinuous” option [1]. This option was used to ensure that the imputation model for the GAC outcomes converged. It works by imputing the GAC outcomes using ordinal regression, but when these outcomes are included as predictor variables in the imputation model for other outcomes they are assumed to be continuous variables, rather than categorical, to reduce the number of degrees of freedom in the imputation model. This model simplification was needed to ensure the imputation model converged and was a reasonable assumption given that the GAC outcomes are measured using a relatively large number of the response categories (six in total). After the imputation model had been applied to the data, Rubin's rules [3] were used to combine treatment effects (and their associated standard errors) across the imputed data sets to provide a single estimate of treatment effect for each analysis outcome.

**References**

[1] StataCorp. 2017. *Stata Statistical Software: Release 15*. College Station, TX: StataCorp LLC.
[2] White IR, Royston P, Wood AM. Multiple imputation using chained equations: issues and guidance for practice. Stat Med 2011;30:377–99. 44.

## Appendix 2 Number of patients treated at each clinic and by each physiotherapist

Figures are numbers unless otherwise stated. ^α^ Physical therapists were defined as treating the patient if they treated the patient for the largest number of treatment sessions, or, when two physical therapists treated a patient for an equal number of sessions, as the physiotherapist that had seen the patient for the first treatment session. ^β^ = One physical therapist delivered four treatment sessions, but they were not frequent enough to be defined as the main physical therapist treating the patient. Abbreviations: ITE, individually tailored exercise; TEA, targeted exercise adherence; UC, usual physical therapy care

## Appendix 3 Primary outcomes at each time-point when adjusting for therapist effects, removing adjusting covariates, and not imputing for missing data

Figures are presented after imputation of missing data and after adjustment for baseline WOMAC scores, age, gender, onset of knee problem, and treatment centre unless otherwise stated. Higher WOMAC scores = worse outcome. The intra-class correlations (ICC) for treating therapist, when adjusted for model covariates, was 0 at all time-points except for WOMAC pain at 6- and 9-month follow-up (ICC = 0.02 and 0.01 respectively). Abbreviations: BL, baseline; CI, confidence interval; ITE, individually tailored exercise; TEA, targeted exercise adherence; UC, usual physiotherapy care; WOMAC, Western Ontario and McMaster Universities Osteoarthritis Index

## Appendix 4 Analysis of the primary outcome at the primary time-point (i.e. the 6-month follow-up) stratified by level of self-reported exercise adherence at 6-month follow-up

^α^ The interaction regression coefficients express the interaction between treatment arm and level of exercise adherence after the outcome of interest has been adjusted in a regression model for baseline in the outcome of interest, age, gender, duration of the knee problem and physiotherapy treatment clinic (as was used in the randomisation algorithm)

53 participants reported they were “not sure” if they had been doing their exercises as often as advised so were excluded from the analysis.
